# More than the sum of its parts: Merging network psychometrics and network neuroscience with application in autism

**DOI:** 10.1162/netn_a_00222

**Published:** 2022-06-01

**Authors:** Joe Bathelt, Hilde M. Geurts, Denny Borsboom

**Affiliations:** Department of Psychology, Royal Holloway, University of London, Egham, Surrey, UK; Department of Psychology, University of Amsterdam, Amsterdam, the Netherlands

**Keywords:** Network, Methods, Psychometrics, Neuroimaging, Autism

## Abstract

Network approaches that investigate the interaction between symptoms and behaviours have opened new ways of understanding psychological phenomena in health and disorder in recent years. In parallel, network approaches that characterise the interaction between brain regions have become the dominant approach in neuroimaging research. In this paper, we introduce a methodology for combining network psychometrics and network neuroscience. This approach utilises the information from the psychometric network to obtain neural correlates that are associated with each node in the psychometric network (network-based regression). Moreover, we combine the behavioural variables and their neural correlates in a joint network to characterise their interactions. We illustrate the approach by highlighting the interaction between the triad of autistic traits and their resting-state functional connectivity associations. To this end, we utilise data from 172 male autistic participants (10–21 years) from the autism brain data exchange (ABIDE, ABIDE-II) that completed resting-state fMRI and were assessed using the autism diagnostic interview (ADI-R). Our results indicate that the network-based regression approach can uncover both unique and shared neural correlates of behavioural measures. For instance, our example analysis indicates that the overlap between communication and social difficulties is not reflected in the overlap between their functional brain correlates.

## Author Summary

The article introduces a method to combine common practices in network psychometrics and network neuroimaging. Namely, we use the unique variance in behavioural measures as regressors to identify unique neural correlates. This enables the description of brain-level and behavioural-level data into a joint network while keeping the dimensionality of the results manageable and interpretable. We illustrate this approach by showing the network of autistic traits and their correlates in resting-state functional connectivity.

## INTRODUCTION

The traditional view of psychiatric conditions conceptualizes mental disorders as latent constructs that are expressed in a set of manifest symptoms. For instance, autism spectrum disorders (ASDs) are typically described as a disorder rooted in an (as yet unknown) dysfunction rooted in the biology of the human system, which manifests its effects in the domains of social interaction, social communication, and restricted, repetitive patterns of behaviour, interests, or activities (DSM-5). In this view, the manifested difficulties—typically interpreted as *symptoms*—are viewed as indicators of a latent condition; in accordance, the information present in these symptoms is commonly aggregated into a single score (e.g., by counting the number of symptoms or averaging subscales of questionnaires). This score is interpreted as a measure of the severity of the mental disorder, and usually functions as a dependent variable in designs geared to uncover the genetic background or neural correlates of the disorder.

An alternative view, which has emerged in recent years, interprets the relation between symptoms and disorders differently, and emphasises the dynamic interaction between symptoms (Borsboom, 2017; Borsboom et al., 2018; Robinaugh et al., 2020). In this alternative *network approach to mental disorders*, causal interaction between symptoms themselves is brought to the forefront and disorders are seen as clustered states of symptoms that mutually reinforce each other. For instance, in the case of autism, restricted interests may limit the time spent in social interactions, which may lead to reduced social communication skills, and the limited success in social communication may in turn reinforce restricted interests. From this perspective, an important part of the basis of mental disorders like autism would be expected to lay in the disposition to develop individual symptoms and in the processes that govern interactions between them. As a result, the network approach does not focus on the level of aggregate scores or functions thereof, but on the patterns of association that arise between symptom variables. A host of statistical techniques has been developed to estimate and analyse such symptom networks (Borsboom et al., 2021). This way of working has become a popular methodology approach to the study of symptomatology and is beginning to find its way in clinical practice (Kroeze et al., 2017).

By focusing attention on the interaction between symptoms, network approaches open up new ways to study how mental disorders may be related to the brain. In particular, from the point of view of network theory, neuroscientific approaches should focus on (a) mechanisms or dysfunctions that lead symptoms to arise (e.g., mechanisms that promote repetitive behaviours), and (b) processes that couple one symptom to another (e.g., mechanisms that link repetitive behaviours to, say, problems in social interaction). To the extent that biological processes are specific to individual symptoms or symptom-symptom interactions, aggregation of scores is methodologically inadvisable because the shared variance between symptoms is then likely to correspond to a biological amalgam of processes and mechanisms that will be hard to tease apart.

Autism spectrum condition (ASC) is an ideal test case for applying the network perspective to resolve ongoing debates about the associations between core behavioural features. ASC is characterised by atypicalities in social interaction and communication, alongside restricted and repetitive behaviours and narrow interests (RRBI). However, there is some debate regarding the separation between the domains. On one hand, it has been noted that atypicalities in all three domains frequently co-occur (Wing & Gould, 1979), possibly reflecting a shared genetic or neurocognitive mechanism. On the other hand, the ‘fractionable triad’ account suggests that unique mechanisms contribute to difficulties in each domain (Happé & Ronald, 2008). Factor analyses of scores from diagnostic interviews or behaviour ratings are inconclusive. Several studies indicate that a large proportion of variance loads onto a single factor in principal component analysis (Constantino et al., 2004; Szatmari et al., 2002), which is interpreted as support for a single factor underlying the triad of autistic behaviours. Yet, other studies found support for a factor structure with two, three, or more factors (Berument et al., 1999; DiLalla & Rogers, 1994; Kim et al., 2019; van Lang et al., 2006; Wadden et al., 1991). A meta-analysis indicated that studies that identified more than one factor, typically divided the social aspects (social interaction, communication) from the putatively less social aspects (RRBIs) (Mandy & Skuse, 2008). However, a more recent study that used a hierarchical cluster model indicated that communication and RRBIs segregated together with social interaction as a separate factor (Kim et al., 2018). In summary, the behavioural evidence suggests that the domains of atypicality in autism (social interaction, communication, RRBIs) are linked but can be fractionated. This provides an ideal test case for a network approach that aims to capture the relation between associated variables.

In addition to investigations at the behavioural level, methodologies developed in the network approach may be useful in disentangling neural correlates on symptoms and interactions between them. In the case of ASC, neuroimaging research has associated the autistic trait triad with partially distinct networks of brain regions. Social cognition associated with the social interaction aspect of the autistic trait triad is thought to be supported by a distributed network of brain regions, collectively referred to as the “social brain” (Müller & Fishman, 2018). This network comprises of the amygdala, the dorsal and ventral medial prefrontal cortices (dmPFC, vmPFC), the anterior and posterior cingulate cortex (ACC, PCC), the posterior superior temporal sulcus (pSTS), the temporoparietal junction (TPJ), the inferior occipital gyrus (IOG), the fusiform face area (FFA), and the insula. These regions showed increased responses in tasks that tap social processing, including the decoding of facial expressions (Kadosh et al., 2010) and theory of mind (Schurz et al., 2014). These social brain regions show reduced activation during social processing tasks in autism (Di Martino et al., 2009; Patriquin et al., 2016). Furthermore, the connectivity between regions of the social regions is weaker in autism, yet the connectivity with regions outside of the social brain is stronger compared to neurotypicals (Hagen et al., 2013; Kana & Wadsworth, 2012; Supekar et al., 2013). Differences in social brain connectivity are also apparent at rest. Assaf et al. (2010) reported reduced connectivity of the canonical default mode (DMN) and salience network (SN) that contain social brain regions. Reduced connectivity of the DMN and SN was related to the severity of social symptoms. Communication atypicalities in autism have been linked to differences in classic language areas, such as Broca’s and Wernicke’s areas. Individuals with autism show reduced activation in the left inferior frontal cortex (Broca’s area) and increased activation in the superior temporal gyrus (Wernicke’s area) in lexical and semantic processing tasks (Harris et al., 2006; Just et al., 2004; Kana et al., 2006). Differences in connectivity within the language network is also apparent at rest. The left inferior frontal cortex (Broca’s area) has been found to show reduced connectivity in children and adolescents with autism, while the superior temporal gyrus (Wernicke’s area) shows reduced connectivity in autistic adults (Y. Lee et al., 2017). Differences related to communication difficulties in autism are also observed outside of the core language network. This includes increased activation of homologous areas in the right hemisphere during language processing (Just et al., 2004; Kana et al., 2006) and activations in areas typically involved in visuospatial processing (Kana et al., 2006; Samson et al., 2012), that is, the lateral occipital cortex and inferior parietal sulcus. Similarly, studies that investigated functional connectivity at rest indicated an association of higher communication difficulties with increased connectivity across the whole brain (Uddin et al., 2013), reduced connectivity of the DMN and SN (Assaf et al., 2010; Maximo & Kana, 2019), and increased connectivity of the lateral occipital cortex (Jung et al., 2017). Repetitive and stereotyped behaviours and narrow interests (RRBIs) have been associated with differences in systems involved in sensation, motor control, and reward-related processing. Specifically, higher RRBIs scores are linked to greater connectivity between the striatum with occipital and frontal areas (Dupong & Di Martino, 2020), alongside lower connectivity of the striatum with cortical motor and sensory areas (Abbott et al., 2018; Maximo & Kana, 2019). Furthermore, higher RRBIs scores are associated with lower connectivity of fronto-parietal regions (Abbott et al., 2018; J. M. Lee et al., 2016), broadly consistent with the DMN. Furthermore, SN hyperconnectivity has been found to predict RRBIs scores in autistic children (Uddin et al., 2013).

As is apparent from the summary above, there is a rich literature on the brain correlates of autistic traits. In some cases, the associations overlap. For instance, the default mode and SN are implicated across all domains of the autistic triad. Other associations appear to be unique to each domain, for example, the involvement of the basal ganglia in RRBIs. The interplay between brain systems that are associated with autistic behaviours may be key. For instance, the involvement of the same brain system may explain the overlap of characteristics at the behavioural level. However, the interaction between autistic traits and their neural correlates has so far not been investigated. A network approach that combines behavioural and brain measures is primed to fill this gap. However, a clear link between the network psychometrics approach and brain imaging research has so far been missing. Establishing this connection has the potential to open a new line of research that investigates the relations between brain-level and symptom-level networks and, thereby, holds the potential to uncover mechanisms of compensation or vulnerability. However, the current approaches in psychiatric neuroimaging are not well suited to this task. The dominant approaches are to either contrast groups of cases with groups of controls, or to identify correlates of symptoms or putative cognitive endophenotypes. Both approaches have limitations as has been discussed at length elsewhere (Borsboom, 2017; Robinaugh et al., 2020). Most importantly for network psychometrics, these approaches implicitly assume that psychiatric conditions are latent constructs that determine, and therefore are indicated by, symptoms or cognitive endophenotypes. In this manuscript, we provide a conceptual bridge that links network psychometrics with established methods in human neuroimaging. Namely, we construct brain-level correlates by using the unique variance in behavioural measures as regressors. Subsequently, we explore the relationship between the symptom-level network and its neural correlates.

To illustrate this approach, we focus on the behaviours associated with ASC. We use large databases that collected resting-state fMRI (rsfMRI) and characterised autistics traits with the same assessment instrument to study the neural correlates of symptom networks. Using these data, we applied the connectome-based predictive modelling (CPM) method (Shen et al., 2017) to identify the rsfMRI correlates of autistic traits. To characterise the relations between behavioural and neuroimaging data, we applied network psychometric and causal inference methods. Based on the psychometric literature on autistic traits summarized above, we expected a closer association between social and communication difficulties than between these traits with RRBIs. We did not have a strong expectation regarding the association between the neural correlates.

## MATERIALS AND METHODS

### Network-Based Regression Method

The following section describes new methods for constructing networks based on brain correlates of behavioural or cognitive measures.

### Behaviour Network–Based Regressors

In neuroimaging, out-of-scanner task performance scores are often used to identify the neural correlates of behaviours, particularly for analyses of resting-state data (fMRI, M/EEG) or structural data (diffusion-weighted imaging, morphological). However, the use of raw task scores has been criticised, because the task scores reflect many extraneous influences. One way to create better regressors is to calculate the shared variance of several measures that are thought to tap the same psychological construct (Poldrack & Yarkoni, 2016). This approach of creating latent variables views the psychological or psychiatric constructs as latent entities indicated by the scores. In contrast, in network psychometrics, one may be more interested in the unique variance of each measure to create the nodes that make up the network. For neuroimaging, obtaining correlates for measures while controlling for other measures is already common practice, typically in the context of controlling for nuisance variables. This can be achieved by simply regressing the effect of other variables from each variable and retaining the residual. For instance, in a network of three variables A, B, and C, the unique variance in A is given by the residual term, epsilon, in the regression equation: *y*_*A*_ = *β*_*B*_*X*_*B*_ + *β*_*C*_*X*_*C*_ + *X*_*Intercept*_ + *ϵ*. As expected, the association between behavioural scores was removed after regressing the effect of the other behavioural scores (see Table 1). The residual terms can be used as regressors to obtain the neural correlates of the unique variance of each behavioural measure. In this way, the nodes within a psychometric network can be used as regressors in established neuroimaging pipelines. This provides a methodological and conceptual bridge between network psychometrics and neuroscience.

**Table 1.** Correlation between ADI-R domain scores for either the original scores (1–3) or the residuals after regression (4–6)

	1	2	3	4	5	6
1. Social	1.00	0.66	0.38	0.73	0.56	0.38
2. Communication	0.66	1.00	0.36	0.00	0.93	0.36
3. RRBI	0.38	0.36	1.00	0.00	0.00	1.00
4. Social residual	0.73	0.00	0.00	1.00	0.00	0.00
5. Communication residual	0.56	0.93	0.00	0.00	1.00	0.00
6. RRBI residual	0.38	0.36	1.00	0.00	0.00	1.00

### Associations Between Behaviour Scales and Functional Brain Connectivity

**Figure 1.**
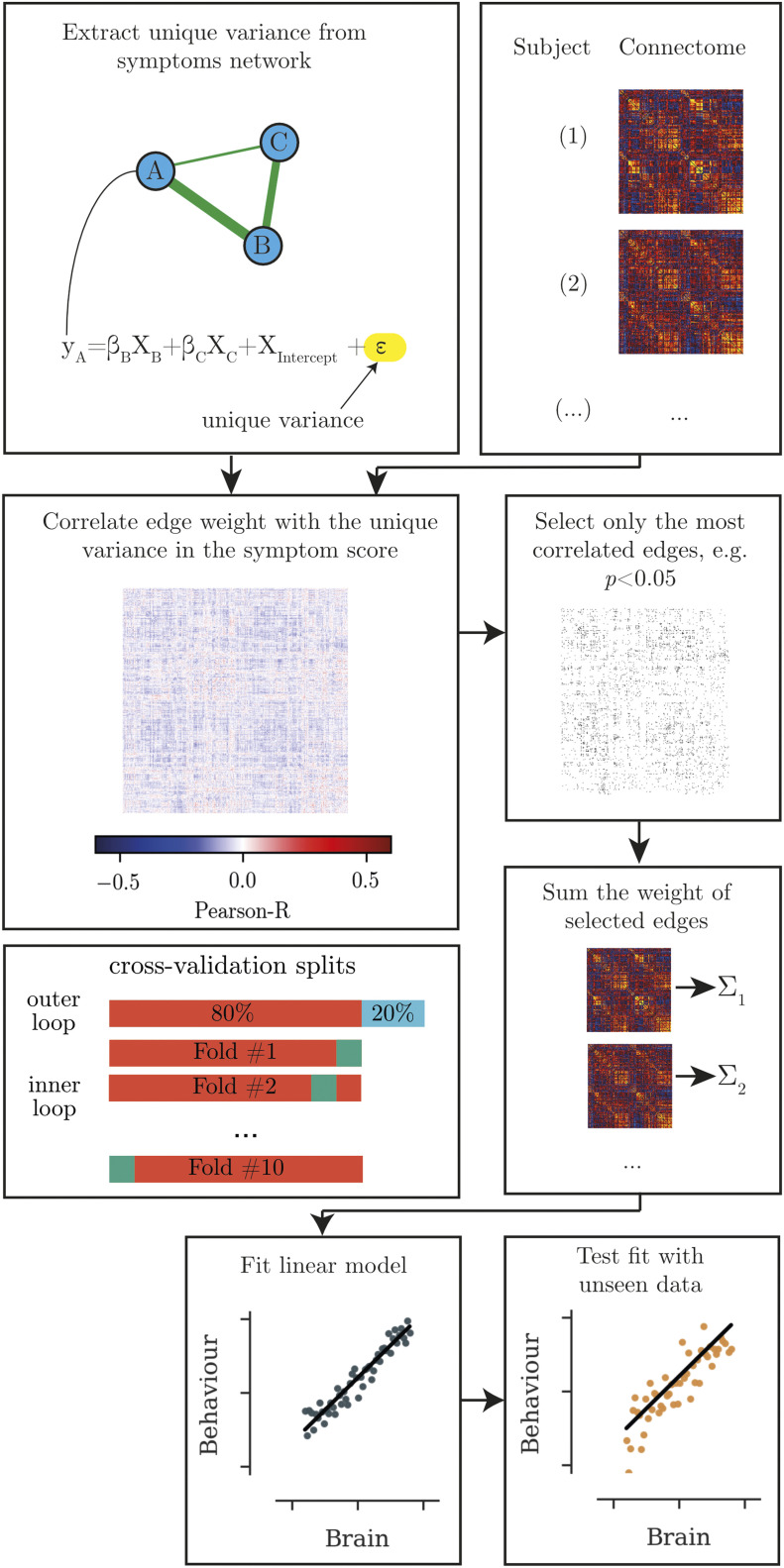
Overview of the analysis steps for identifying the brain correlates of behavioural measures. First, the unique variance in each behavioural scale is calculated. The edges that shows the strongest correlation with the unique variance in the behavioural score are extracted. The summed edge weight of the most highly associated edges is used to build a regression model, which is then tested in unseen data.

To identify the associations between functional connectivity and behavioural ratings, we used the CPM approach described by Shen et al. (2017) (see Figure 1 for an overview). In short, a set of edges is identified that correlate with the behaviour ratings below a certain *p* value threshold. Then, the edge weights are summed into a brain score and entered into a regression model to estimate the association between the brain score and behaviour rating. For the current analysis, we identified positively and negatively associated edges separately and entered the summed edge weight for positively and negatively associated edges into a common multiple regression model:

$$y_{\text{symptom}}=\beta_{\text{Brai}n^{+}}X_{\text{Brai}n^{+}}+\beta_{\text{Brai}n^{-}}X_{\text{Brai}n^{-}}+X_{\text{Intercept}}+\epsilon$$

**Figure 2.**
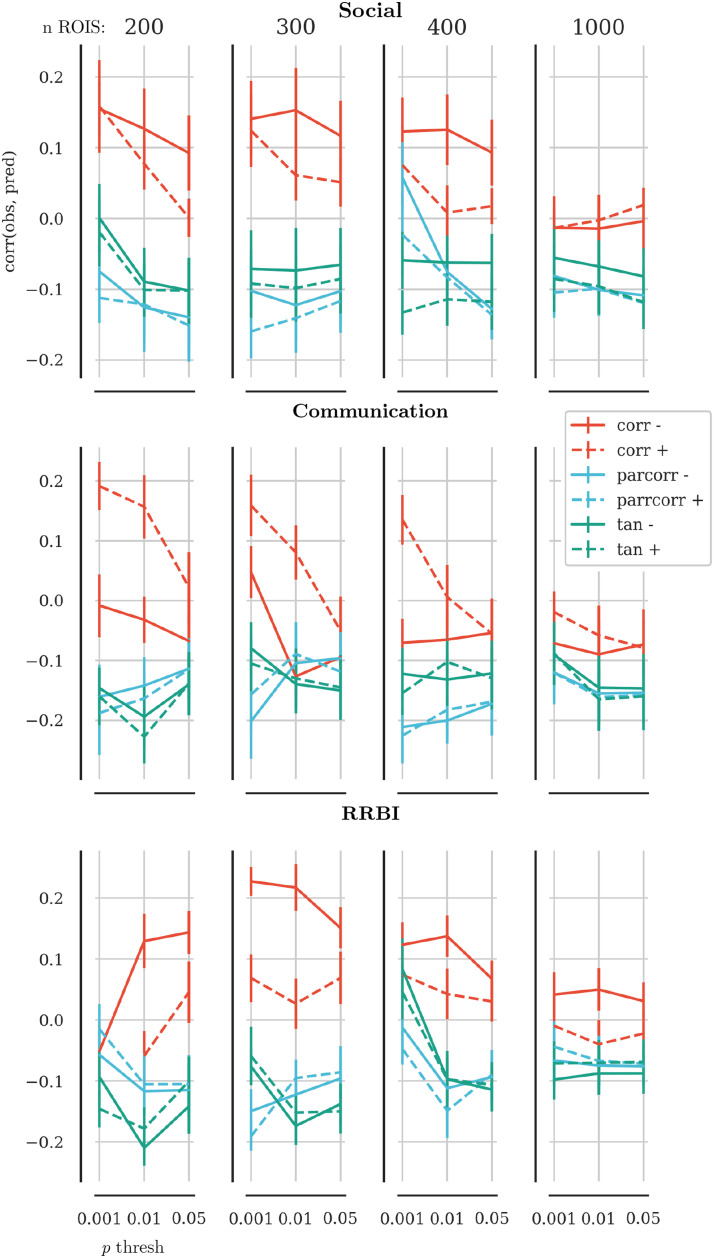
Results of the parameter tuning. The *x*-axis shows the *p* value threshold used to select edges that were associated with behaviour ratings scores. The *y*-axis shows the correlation between the predicted scores and the observed scores in held-out data. Solid lines show results based on connectomes that were constructed without global signal regression; dashed lines indicate results based on connectomes with global signal regression. Red lines show results based on connectomes that used Pearson correlation as the edge definition, blue lines show the results for partial correlations, and green lines for tangent-space embedding. The panel in each row show the results for brain parcellations with 200, 300, 400, and 1,000 ROIs. The overall best results across behaviour scales were observed for 300 ROIs.

We were faced with multiple methodological choices that were difficult to determine a priori, namely the number of regions of interest (ROIs) in the parcellation, the edge definition, *p* value threshold, and global signal regression strategy. Therefore, we employed a shuffle split cross-validation to find the combination of parameters that led to the best prediction of behaviour ratings in unseen data. For this parameter tuning, we randomly split the data into an 80% training and 20% test set in an outer loop (see Figure 1 for an illustration of the cross-validation splits). We identified the associated edges and fitted the regression model in the training set using 10-fold cross-validation. Then, we compared the quality of prediction by using the model with the held-out test data from the outer loop by calculating the correlation between predicted and observed values (Shen et al., 2017). The parameter combination that produced the best prediction in unseen data used 300 ROIs, defined the edge weight through Pearson correlation without global signal regression, and set the association *p* value threshold to *p* < 0.001 (see Figure 2 for results of the parameter tuning). For the evaluation of the final model, we only retained edges that were included across 7 out of 10 splits of the data. We compared the performance of this model against 5,000 random permutations of the behavioural data.

### General Data Preparation

The following section describes the data and preprocessing steps that were used to illustrate the network-based regression approach.

### Participants

The analysis was based on data taken from the first and second Autism Brain Imaging Data Exchange database (ABIDE; Di Martino et al., 2014; and ABIDE-II; Di Martino et al., 2017). Both databases collated resting-state fMRI and phenotypic data from autistic participants from 19 international sites. There was no prior coordination between sites, which means that the sites differed in their fMRI acquisition protocols and diagnostic procedures. Because of the ensuing variability, we applied selection criteria to arrive at a more homogeneous sample. Namely, we selected only male participants, because women were not well represented. Furthermore, we selected only participants over 10 years of age, because of different scoring criteria on the ADI-R for younger children. We further excluded participants older than 21 years, because the relatively few participants older than 21 years were spread over a large age range. In addition, we only selected participants with complete, research-reliable ADI-R assessments and with complete structural and functional MRI that was rated as useable by expert human assessors (note that the quality ratings are distributed with the phenotypic data). For further quality control, we excluded 13 participants because their fMRI data fell outside the recommended range on established quality metrics (framewise displacement > 0.5 mm; Power et al., 2012; DVARS > 5%; Burgess et al., 2016). The final sample consisted of 172 autistic participants (ABIDE: *n* = 127, ABIDE-II: *n* = 45; see Table 2 for sample characteristics). Please note that we were not aiming to obtain a representative sample. The purpose of the analysis was to demonstrate the potential use of a network-based regression method. The selection criteria were intended to create a more homogeneous sample with good quality imaging data.

**Table 2.** Sample characteristics

	Mean ± SE	Min-Max	*N* [%]
Age	13.87 ± 0.204	10.04–20.09	
Full-scale IQ^a^	108.94 ± 1.015	86–149	
ADI-R^b^			
Social	19.59 ± 0.388	11,202	164 [95.35]
Verbal	15.51 ± 0.316	45,505	167 [97.09]
RRBI	5.94 ± 0.196	0–12	142 [82.56]

^a^ Assessments: 86 WASI, 53 WISC-IV, 33 other.

^b^ Cutoff scores: Social > 10, Verbal > 8, RRBI > 3.

### Assessment of Autism Characteristics

The Autism Diagnostic Interview-Revised (ADI-R) is a standardised diagnostic interview for primary caregivers (Rutter et al., 2003). It focuses on a description of a child’s behaviour when they were 4–5 years old and their current behaviour. An autism diagnosis is made with an ADI-R algorithm that consists of 37 extracted items. In addition to a total score, subscores for autistic traits (social interaction, communication, RRBI) can be obtained. The ADI-R shows a high interrater agreement (0.94–0.96; Cicchetti et al., 2008) and high convergence with clinical team assessments and another commonly used assessment protocol (75% agreement; Mazefsky & Oswald, 2006), that is, the autism observation schedule (ADOS). The ADI-R protocol is adjusted depending on the chronological and mental age of the participant. Fewer items are included for children younger than 10 years or with an intellectual functioning outside of the typical range. Because the current analysis aimed to compare associations of scores, we restricted our analysis to children above 10 years with a full-scale IQ in the typical range to ensure that differences in ADI-R scores reflected the relative ranking of the severity of difficulties in each domain. Please note that only summary scores for each domain (social interaction, communication, RRBI) were part of the ABIDE data release, that is, the individual items used to calculate the summary scores were not available for analysis.

### fMRI Processing

The data were processed using the standard configuration of the Configurable Pipeline for the Analysis of Connectomes (C-PAC, v. 1.6.2; Craddock et al., 2013). C-PAC is an automated state-of-the-art pipeline for reproducible processing of large-scale data. The current analysis was run using the singularity image distributed via the C-PAC website to process the data on a high-performance computing cluster. The configuration file for the pipeline is provided via the Open Science Framework so that the results can be exactly reproduced (https://osf.io/my8g6/?view_only=85c585b0f4ee4abb8e700afd2693b8c3). The full details of the processing pipeline are available from the C-PAC website (https://fcp-indi.github.io/docs/user/preprocessing). The following provides an overview of the preprocessing steps. For anatomical preprocessing, a nonlinear transform between images and a 2-mm MNI brain-only template were calculated using Advanced Normalisation Tools (ANTs) (Tustison et al., 2014). The images were then skull-stripped using AFNI’s 3dSkullStrip and subsequently segmented into white matter (WM), grey matter (GM), and cerebrospinal fluid (CSF) using FSL’s fast tool (Smith, 2002). The resulting WM mask was multiplied by a WM prior map that was transformed into individual space using the inverse of the linear transforms calculated through ANTs. A CSF mask was multiplied by a ventricle map derived from the Harvard–Oxford atlas distributed with FSL (Smith et al., 2004). Skull-stripped images and GM tissue maps were transformed into MNI space at 2-mm resolution.

For functional preprocessing, motion correction was performed using a two-stage approach in which the images were first co-registered to the mean of the fMRI sequence, and then a new mean was calculated and used as the target for a second registration (AFNI 3dvolreg; Cox & Jesmanowicz, 1999). A 7-degree of freedom linear transform between the mean fMRI and the structural image was calculated using FSL’s boundary-based registration (Greve & Fischl, 2009). Nuisance variable regression (NVR) was performed on the motion-corrected data using a second-order polynomial, a 24-regressor model of motion (Friston et al., 1996), five nuisance signals identified via principal components analysis of signals obtained from WM (CompCor; Behzadi et al., 2007), and the mean CSF signal. WM and CSF signals were extracted using the previously described masks after transforming the fMRI data to match them in 2-mm space by using the inverse of the linear fMRI-sMRI transform. The NVR procedure was performed twice, with and without the inclusion of the global signal as a nuisance regressor. The results of the processing strategies were both entered into the predictive model in the later stages of the analysis (see below). The residuals of the NVR procedure were bandpass filtered (0.001 Hz < f < 0.1 Hz), written into MNI space at 2-mm resolution and subsequently smoothed using a 6-mm full-width half-maximum kernel.

Then, the time series for the parcellation described by Schaefer et al. (2017) were extracted with 200, 300, 400, and 1,000 parcels. We chose this parcellation because it provides a better account of fMRI activations than previous data-driven parcellations and is based on a large representative database (*N* = 1,489). We evaluated the optimal resolution for the purpose of the current analysis in the predictive model. To ensure that all included regions were sufficiently covered in all participants, we calculated the mean functional image, thresholded and binarized it at 70% intensity, and calculated the overlap between the atlas ROIs and the resulting image in all participants. ROIs that had less than 50% overlap with the binarised intensity image in any participant were excluded from the analysis. This resulted in the exclusion of 9 ROIs for the 200 ROI atlas, 16 for the 300 ROI atlas, 24 for the 400 ROI atlas, and 68 for the 1,000 ROI atlas. The excluded ROIs were located in the frontal and temporal pole.

### Functional Connectome Construction

We calculated the functional connectome matrices using three approaches, namely correlation, partial correlation, and tangent-space embedding. For the correlation approach, we calculated the Pearson correlation between each pairwise combination of ROIs in the parcellation. For the partial correlation approach, the Pearson correlation of each pairwise combination of ROIs was calculated after regressing the effect of other ROIs from both time series. For the tangent-space embedding approach, we used the method described by Varoquaux et al. (2010) that models each participant as a deviation from the group average connectome. For all approaches, the implementation of the method in Nilearn v. 0.6.2 was used (Abraham et al., 2014).

To reduce the influence of extraneous variables, we applied a similar regression approach to the Human Connectome Project Mega-Trawl analysis (https://db.humanconnectome.org/megatrawl/HCP820_MegaTrawl_April2016.pdf). Namely, we regressed the effect of age, age^2^, acquisition site, framewise displacement, spatial root-mean-square of the data after temporal differencing (DVARS), intracranial volume (ICV), and total GM volume from each edge in the functional connectome in an ordinary least-squares regression model. ICV and total GM volume were estimated using FreeSurfer.

### Quality Control

Functional MRI connectomics have been shown to be particularly sensitive to participant movement (Power et al., 2012). In order to mitigate the influence of motion, we applied a combination of steps. First, we only included participants with structural and functional data that were rated as useable by expert human assessors. These assessments are included in the phenotypic data file of the ABIDE and ABIDE-II database. For some sites, scans were assessed by multiple assessors. We only included a participant if all assessors rated the data as useable. Furthermore, we evaluated the functional data using metrics calculated using the MRIQC pipeline (Esteban et al., 2017). We only included data that met conservative thresholds for framewise displacement (<0.5 mm; Power et al., 2012) and DVARS (<5%; Burgess et al., 2016). In the remaining participants, the effect of participant motion was mitigated by controlling for nuisance signals, including noise components estimated in the WM (Behzadi et al., 2007). Furthermore, we regressed mean framewise displacement and standardised DVARS from each edge in the functional connectome. The quality metrics were not associated with social and communication scores (all *p* > 0.16 uncorrected). RRBI scores were associated with DVARS (*p* = 0.032 uncorrected), but not framewise displacement (*p* = 0.214 uncorrected). We compared the effect of different de-confounding strategies as implemented in the confounds package for Python (Raamana, 2020) using cross-validation. Regressing the effect of confounding variables showed the numerically highest prediction but the differences between confounding strategies were negligible (augment: mean = 0.008, *SE* = 0.0011; dummy: mean = 0.008, *SE* = 0.0011; residualize: mean = 0.009, *SE* = 0.0011, *F*(2, 31638) = 0.62, *p* = 0.54; all values indicate correlations between predicted and observed scores in unseen data). Confound regression was employed for confound removal for the final analysis.

### Interpretation of Imaging Results

To aid the interpretation of the neuroimaging associations, we obtained anatomical labels for the centroid coordinates of the nodes that were implicated in the association between rsfMRI connectivity and behavioural measures. For this purpose, we used FSL’s *atlasquery* tool with the Harvard–Oxford Cortical Structural atlas. We report most likely labels for each centroid coordinate.

### Construction of the Behaviour and Brain Score Network

To assess the relations between behavioural scores and their neural correlates, we estimated the network structure of a network based on the behavioural scores and the total brain score for each behavioural measure. To construct the total brain score, the regression weights were applied to the positive and negative brain scores. To determine the edges of the network, we identified an unregularized Gaussian graphical model by minimizing the extended Bayesian information criterion (BIC) using the glasso algorithm and stepwise model selection. These analyses were performed in R (version 3.5.0) using the *bootnet* v1.4.3 and *qgraph* v1.6.5 packages.

Subsequently, we identified the likely causal direction of the network edges (Pearl, 2014) in the network of behavioural scores, rsfMRI correlates, and a combined network with behavioural and rsfMRI correlate notes. For this purpose, we computed a Bayesian network, described in a directed acyclic graph (DAG), using structure learning algorithms implemented in the *bnlearn* v4.5 package (Scutari, 2009). The structure learned by these algorithms can contain directed and undirected edges. We compared the structure learned through different algorithms, namely the *Grow-Shrink* (Margaritis, 2003), *Incremental Association* (Tsamardinos et al., 2006), *Fast Incremental Association* (Yaramakala & Margaritis, 2005), *Interleaved Incremental Association* (Tsamardinos et al., 2003), and *Max-Min Parents and Children* (Tsamardinos et al., 2006) algorithms. We also compared the structure learned when using different independence test statistics, namely *Pearson correlation*, *Fisher’s Z*, *Monte-Carlo permutation*, and *mutual information*.

## RESULTS

The CPM methods indicated a combination of edge weights that could predict behaviour scores in held-out data (Social: mean = 0.14, *SE* = 0.054; Communication: mean = 0.05, *SE* = 0.043, RRBI: mean = 0.23, *SE* = 0.024; mean correlation between observed and predicted scores across 10 random 80/20 splits) and were significantly better in predicting behaviour scores compared to scrambled data (Social: *p* = 0.021; Communication: *p* = 0.014, RRBI: *p* = 0.011; *p* value based on 5,000 permutations).

**Figure 3.**
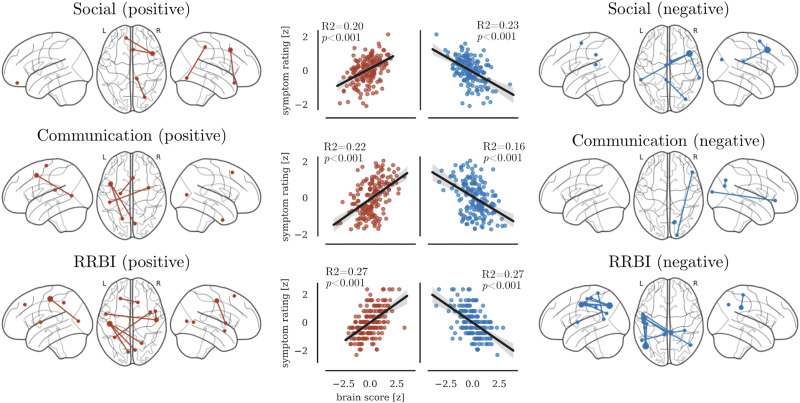
Association between unique variance in ADI-R domains with edges of the functional connectome identified through CPM. The left panel shows edges that were positively associated with scores. The right panel shows negatively associated edges. The scatter plots show the association between the summed brain score and the symptom ratings across the entire sample.

Social difficulties were positively associated with connections of the right prefrontal cortex, and connections between the left fusiform gyrus and right postcentral gyrus (see Figure 3 and Table 3). Furthermore, social difficulties were negatively associated with connections between the right middle frontal cortex with parietal areas in the left and right hemisphere (see Figure 3 and Table 3). Communication difficulties were positively associated with connections between parietal areas in the left hemisphere with medial frontal and occipital areas (see Figure 3 and Table 3). Negative association between communication scores and rsfMRI connectivity were found for connections between the right occipital areas with the inferior frontal gyrus and precuneus (see Table 3). RRBI scores showed the most extensive associations. RRBI scores were positively associated with connections between the left postcentral gyrus with occipital areas, and between the right postcentral gyrus with the left insula. RRBI scores were negatively associated with connections between left pre- and postcentral gyrus with occipital and posterior cingulate areas (see Figure 3 and Table 3).

**Table 3.** Detailed description of edges that were associated with ADI-R scores

	MNI			Harvard-Oxford Label	MNI			Harvard-Oxford Label
Social: positive	−10	48	−22	L Frontal Pole (45%)	44	16	46	R Middle Frontal Gyrus (60%)
	28	−74	−12	R Occipital Fusiform Gyrus (67%)	12	−36	52	R Postcentral Gyrus (34%)
	4	24	−22	R Subcallosal Cortex (59%)	44	16	46	R Middle Frontal Gyrus (60%)
Social: negative	−56	−38	16	L Juxtapositional Lobule Cortex (70%)	44	16	46	R Middle Frontal Gyrus (60%)
	−4	−8	60	L Planum Temporale (48%)	44	16	46	R Middle Frontal Gyrus (60%)
	−4	−38	36	L Cingulate Gyrus posterior division (81%)	36	−78	26	R Lateral Occipita (53%)
	64	−34	22	L Supramarginal Gyrus posterior division (22%)	44	16	46	R Middle Frontal Gyrus (60%)
	6	0	64	R Juxtapositional Lobule Cortex (58%)	44	16	46	R Middle Frontal Gyrus (60%)
Communication: positive	−18	−64	6	L Intracalcarine Cortex (48%)	−42	8	48	L Middle Frontal Gyrus (53%)
	−44	−30	18	L Parietal Operculum Cortex (67%)	36	0	−44	R Temporal Fusiform Cortex (44%)
	−22	−12	68	L Precentral Gyrus (31%)	4	20	54	R Superior Frontal Gyrus (62%)
	−42	8	48	L Middle Frontal Gyrus (53%)	8	−74	8	R Intracalcarine Cortex (62%)
Communication: negative	18	−98	14	R Occipital Pole (60%)	50	32	−4	R Inferior Frontal Gyrus (30%)
	12	−72	26	R Cuneal Cortex (43%)	14	−70	38	R Precuneous Cortex (35%)
RRBI: positive	−6	−78	8	L Intracalcarine Cortex (64%)	−44	−20	54	L Postcentral Gyrus (37%)
	−44	−20	54	L Postcentral Gyrus (37%)	8	−74	8	R Intracalcarine Cortex (62%)
	−44	−20	54	L Postcentral Gyrus (37%)	22	−60	6	R Intracalcarine Cortex (35%)
	−34	−48	46	L Superior Parietal (38%)	40	4	−12	R Insular Cortex (59%)
	−52	0	6	L Central Opercular (63%)	52	−12	50	R Postcentral Gyrus (52%)
	−22	32	42	L Superior Frontal (44%)	14	24	60	R Superior Frontal (61%)
	52	−12	50	R Postcentral Gyrus (52%)	50	6	4	R Central Opercular (37%)
	50	6	4	R Central Opercular Cortex (37%)	52	−12	50	R Postcentral Gyrus (52%)
RRBI: negative	−50	−8	42	L Precentral Gyrus (44%)	−46	−66	40	L Lateral Occipital Cortex (70%)
	−50	−8	42	L Precentral Gyrus (44%)	−4	−38	36	L Cingulate Gyrus (81%)
	−50	−8	42	L Precentral Gyrus (44%)	46	−64	42	R Lateral Occipital Cortex (62%)
	−50	−16	44	L Postcentral Gyrus (46%)	−46	−60	24	L Angular Gyrus (50%)
	−50	−16	44	L Postcentral Gyrus (46%)	−46	−66	40	L Lateral Occipital Cortex (70%)
	−48	−28	56	L Postcentral Gyrus (55%)	−46	−66	40	L Lateral Occipital Cortex (70%)
	−44	−20	54	L Postcentral Gyrus (37%)	−46	−66	40	L Lateral Occipital Cortex (70%)
	−8	−42	66	L Postcentral Gyrus (47%)	−54	−54	12	L Middle Temporal Gyrus (33%)
	−52	0	6	L Central Opercular Cortex (63%)	6	−38	36	R Cingulate Gyrus (70%)
	34	−34	62	R Postcentral Gyrus (44%)	6	−38	36	R Cingulate Gyrus (70%)

*Note*. The MNI coordinates refer to the centroid of the ROI in the Schaefer parcellation with 300 ROIs at 2-mm resolution. The labels indicate the highest probability labels in the Harvard-Oxford Cortical Structural atlas. L = left; R = right.

### Combined Behaviour and Brain Network

**Figure 4.**
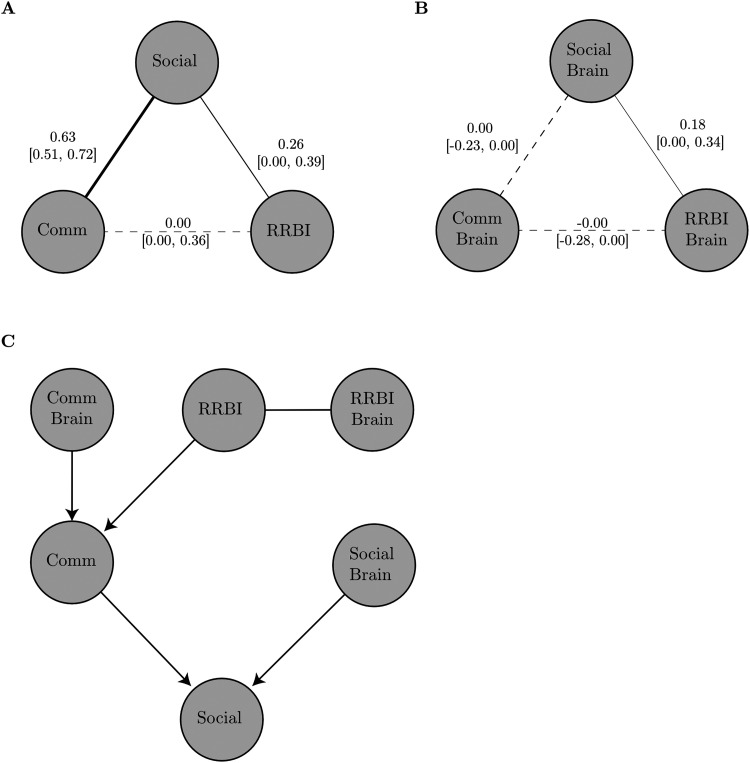
Overview of the network analysis. Bootstrapped undirected network structure of the behavioural measures (A) and their resting-state fMRI correlates (B). (A) Bootstrapped undirected network structure. Solid lines indicate significant edges; dashed lines indicate nonsignificant ones. The thickness of the lines indicates the strength of the association. All edges are positive and solid lines indicate significant edges (bootstrap *p* value < 0.05). The numbers indicate the bootstrapped median with the 2.5% and 98.5%ile in brackets. (C) Directed acyclic graph determined through Bayesian network analysis with constrained-based learning algorithms for the combined brain–behaviour network. Arrows indicated directed interactions. Lines indicate undirected interaction.

We compared the network structure of a network based on behavioural measures and a network based on the neural correlates of the behavioural measures (see Table 4 for correlations between all measures). As expected from previous studies, the behavioural network showed a strong association between Communication and Social Scores, and a weaker association between Social and RRBI scores (see Figure 4A). In contrast, the network of rsfMRI correlates showed a strong association between the functional brain correlates of Social and RRBI scores, but no association between the correlates of Social and Communication scores (see Figure 4B).

**Table 4.** Pearson correlation between behaviour and brain nodes

	1	2	3	4	5	6
1. Social	1.00					
2. Comm	0.66	1.00				
3. RRBI	0.38	0.36	1.00			
4. Soc Brain	0.49	0.09	0.12	1.00		
5. Comm Brain	0.21	0.47	−0.12	−0.05	1.00	
6. RRBI Brain	0.24	0.18	0.61	0.18	−0.13	1.00

*Note*. Comm = communication; RRBI = repetitive and/or restricted behaviours and narrow interests; Soc = social.

Next, we conducted a causal inference analysis using Bayesian networks. For the network of behaviour scores, there were undirected interactions between all behaviour domains, that is, Social, Communication, and RRBI. This structure was indicated across all structure learning algorithms and statistics for the independence tests. For the network of brain correlates, a structure with an undirected interaction between rsfMRI correlates of Social scores and RRBI scores was indicated. There were no connections with the rsfMRI correlates of the Communication score. This structure was consistent across structure learning algorithms and independence test statistics. When combining the behaviour and brain scores in one causal inference network, a more complex network structure emerged (see Figure 4C). In this network, RRBI behavioural scores predicted Communication behaviour scores, which in turn predicted Social behaviour scores. Social and Communication behaviour scores were also predicted by their rsfMRI correlate scores. This structure was consistent across algorithms and independence test statistics.

## DISCUSSION

In this article, we present a novel approach to study the interaction between behavioural variables and their neural correlates. We demonstrate this approach by investigating the relationship between autistic traits and their resting-state functional connectivity correlates identified through CPM (Shen et al., 2017). Our results indicate that the network of associations between behavioural measures of autistic traits is not the same as the network of their neural correlates. We further demonstrate that complex causal interactions become apparent when behavioural traits and their neural correlates are considered together. This shows that the network-based regression approach can provide new insights into otherwise inaccessible aspects of brain–behaviour associations.

To determine if the results obtained through the CPM method with network-based regressors were reasonable, we compared the findings to the results obtained using the traditional approach to studying brain–behaviour relationships. Regarding the connections implicated in each domain of the autistic traits, the current analysis indicated that lower connection strength of the connections with the middle frontal cortex were associated with higher difficulties across all autistic trait domains. This result is consistent with other reports that suggest a central role of frontal networks in autism (Lin et al., 2019; Lynch et al., 2017), possibly due to a shared influence of executive function difficulties mediated by the frontal cortex on all domains of autistic traits (Yerys et al., 2019). Furthermore, weaker connections of lateral parietal areas were associated with greater difficulties across domains. Hypoconnectivity of parietal areas that form part of the default mode network are among the most consistent findings in autism neuroimaging research (Padmanabhan et al., 2017). In conclusion, the findings using the CPM method with network-based regressors are in line with previously implicated neural correlates of autistic traits.

Critically, our analysis using network-based regressors also identified brain correlates that were shared between only some of the behavioural traits. Communication and RRBIs were both associated with greater connectivity of occipital areas. These connectivity differences may be related to sensory oversensitivity (Green et al., 2016) or increased reliance on visuospatial processing strategies (Kana et al., 2006; Samson et al., 2012). Furthermore, higher connectivity of left postcentral gyrus was associated with both higher difficulties with social interaction and RRBIs. Because the network-based regressors only contain the unique variance in each measure, any remaining overlap hints at a shared mechanism at the brain level.

Our results also indicated unique associations for each domain of autistic traits. Because the network-based regressors remove the overlap between the behavioural measures, these findings suggest unique brain-level mechanisms. For instance, higher connectivity of the precentral gyrus was only associated with RRBIs but not the other autistic traits. This illustrates the utility of the network-based regression approach to identify unique mechanisms of behavioural traits.

The results obtained through network-based regression can be further employed to compare the network structure at the behavioural and brain level. This provides further indication about unique or shared mechanisms at each level of observation. In our autism example, there was a closer association between social and communication scores than between these scores and the RRBI score at the behavioural level. This result is consistent with the distinction between “social” and “nonsocial” traits suggested by some psychometric and genetic studies (Mandy & Skuse, 2008; Ronald et al., 2005) that is now incorporated in DSM-5. However, a different structure was indicated for the network of the rsfMRI correlates of the autistic traits. In the brain score network, correlates of social scores were associated with the correlates of RRBI scores, but not with the correlates of communication scores. This suggests that the close association of social and communication scores at the behavioural level is not due to a shared mechanism in brain function. In contrast, the association between the neural correlates of social and RRBI scores indicates that there is at least some overlap in the brain functional systems underlying these behaviours. We further explored causal interaction within the networks. This analysis indicated complex causal interactions between behavioural measures of autistic traits and their neural correlates that could not be predicted from the network at either the behavioural or functional brain level alone. This causal network indicated that Communication scores mediate the association between RRBI and Social scores when accounting for the overlap in the associated functional brain systems. This shows that the network-based regression approach opens possibilities for analyses that contrast behavioural and brain-level associations to identify potentially shared mechanisms by using the information at the behavioural level to minimize any overlap in the regressors.

To construct a network of neural correlates of behavioural traits, we had to address several methodological challenges. Namely, in the first step of the analysis, we identified the functional connectivity correlates of the unique variance in autistic traits in a large, openly accessible database. The principal challenge here is the high dimensionality of the functional connectivity data. Even with a relatively coarse parcellation of the brain, the number of features by far exceeds the number of participants. For instance, a parcellation with 100 ROIs produces 4,950 features, assuming that the investigator excluded the diagonal and identical features in a symmetric adjacency matrix. Several methods have been developed to tackle this problem. These methods include complex machine learning pipelines, for example, HPC Netmats Metatrawls (https://db.humanconnectome.org/megatrawl/index.html), and dimensionality reduction approaches, such as partial least-squares analysis (Krishnan et al., 2011) or canonical correlation analysis (Zhuang et al., 2020). In the current analysis, we employed the CPM method (Shen et al., 2017) for its relative simplicity and clear interpretability (Scheinost et al., 2019). This method identifies functional connectome features that are most closely associated with the behavioural variable of interest and mitigates overfitting through straightforward cross-validation. Despite its simplicity, the method has been successfully employed to identify functional connectome correlates in several studies (Beaty et al., 2018; Finn et al., 2015; Rosenberg et al., 2016, 2018). Even though the modelling approach is relatively simple, the impact of several methodological choices still needs to be evaluated. Our results show that the choice of the functional connectivity metric, the resolution of the parcellation scheme, and the inclusion of global signal regression strongly impact on the strength of association between the functional connectivity features and the behavioural measures (see Figure 2). We recommend that researchers who wish to apply the brain–behaviour network approach evaluate and report the impact of these methodological choices on the strength of the brain–behaviour association.

The results of the current analysis show that CPM can identify functional connections that are associated with each facet of the autistic trait triad. The summed brain scores significantly predicted the behavioural ratings and explained between 20% and 27% of the variance. The proportion of explained variance is lower than in studies that predicted cognitive task performance (Rosenberg et al., 2016) but is similar to the explained variance in a large-scale study with questionnaire measures (https://db.humanconnectome.org/megatrawl/index.html). Measurement considerations aside, the explained variance in the current analysis may also be lower due to the variability in the dataset. We utilised data from ABIDE and ABIDE-II, which are retrospective collections that were acquired without prior harmonisation of the protocol (Di Martino et al., 2014, 2017). Consequently, there is considerable variation between the acquisition sites (He et al., 2020). We reduced this variability by applying stringent criteria for participant inclusion and by regressing the effect of extraneous variables from the connectome edges, but the remaining unaccounted variance is likely to have impacted on the amount of variance that could be explained in the behavioural measures.

It has to be noted that the network-based regression method has some limitations. First, relatively large samples are needed to obtain stable estimates of the networks. We used summed scores in our example to reduce the dimensionality of the behavioural measures. However, this involves a trade-off. Analyses with more detailed measures show that there is considerable variation within each domain at the behavioural level. For instance, RRBIs are dissociable into factors of repetitive behaviours and insistence on sameness (Szatmari et al., 2006). Summing these dissociable domains may impact on the network structure at the behavioural level and influence subsequent analyses. Researchers will need to decide on the appropriate degree of granularity in the behavioural measures when employing the network-based regression approach for their research questions. Furthermore, the current analysis pipeline was constructed to maximise the correspondence between resting-state fMRI connectivity and behavioural scores. Therefore, we selected the parameters that maximised the correlation between the measures and used cross-validation to prevent overfitting. Future researcher may wish to investigate the impact of the parameter choices, for example, through specification curve analysis.

To conclude, we present a novel method to investigate the network structure of behavioural measures and their neural correlates. This method uses the unique variance int the behavioural variables to identify neural correlates. Subsequently, the behavioural and neural variables can be treated as nodes to compare the network structure at the level of behaviour and brain function. We show how this approach can be used to investigate if similarities at the behavioural level may be driven by similar mechanisms at the functional brain level. The approach presented here aligns closely with the shift towards complex system analysis in clinical psychology (Borsboom et al., 2018) and expands the current toolkit to levels of analysis beyond behavioural traits.

## AUTHOR CONTRIBUTIONS

Joe Bathelt: Conceptualization; Formal analysis; Methodology; Software; Visualization; Writing – original draft; Writing – review & editing. Hilde M. Geurts: Conceptualization; Supervision; Writing – original draft; Writing – review & editing. Denny Borsboom: Conceptualization; Methodology; Supervision; Writing – original draft; Writing – review & editing.

## FUNDING INFORMATION

Joe Bathelt, Amsterdam Brain & Cognition Talent Grant, 2019. Hilde M. Geurts, VICI Grant from the Netherlands Organisation for Scientific Research, Award ID: 453-16-006. Denny Borsboom, Consolidator Grant from the European Research Council, Award ID: 647209.

## TECHNICAL TERMS

RRBI: Repetitive and restricted behaviours or interests; potential domain of autism spectrum condition behaviours.
CPM: Connectome-based predictive modelling; approach to establish an association between connectome measures and behavioural measures.
ADI-R: Autism Diagnostic Interview–Revised; assessment for behaviours associated with autism spectrum condition.
DAG: Directed acyclic graph; directed graph that never forms a closed loop.
